# Surgical management of a massive choledochal cyst

**DOI:** 10.1093/jscr/rjab006

**Published:** 2021-02-14

**Authors:** Atta Nawabi, Javaneh Jabbari, Perwaiz Nawabi

**Affiliations:** The University of Kansas, Department of Surgery, Kansas City, KS 66160, USA; The University of Kansas, Department of Surgery, Kansas City, KS 66160, USA; Kansas City University, Kansas City, MO 64106, USA

## Abstract

Choledochal cysts (CC) are congenital bile duct anomalies, typically present in children. The size of CC vary, but they rarely exceed 9 cm. Surgical resection is the mainstay of treatment. This case report presents 18-year-old female with jaundice and abdominal pain. On imaging she was found to have a type I CC versus a type IVa CC. She was taken to the operating room where she was found to have a 20 cm type I CC. The patient experienced complete recovery after total resection of the extrahepatic cyst with reconstruction with a Roux-en-Y hepaticojejunostomy. Preoperative diagnosis of the type of CCs can be challenging. Proper imaging preoperatively can aid in diagnosis of these cysts, but delineation of anatomy and type may not always be possible. If treated in a timely manner, it can help prevent both long- and short-term complications.

## INTRODUCTION

Choledochal cysts (CC) are rare congenital anomalies of the biliary ducts. More commonly found in children, the incidence ranges from 1 in 13 000 to 1 in 2 million births [1]. Approximately 85% of children present with two of the three symptoms of right upper quadrant mass, jaundice or intermittent colicky abdominal pain. In comparison, ~85% of adults present with only one out of the three symptoms [1]. As a result, cases can often go unrecognized or misdiagnosed in the adult population.

**Figure 1:**
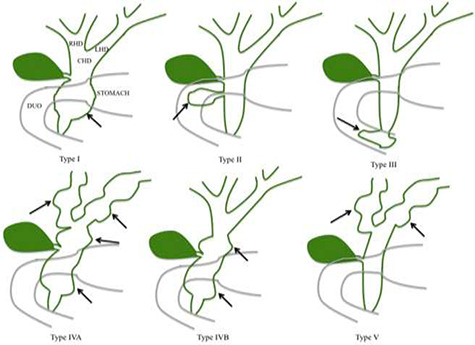
Todani classification of CC.

**Figure 2:**
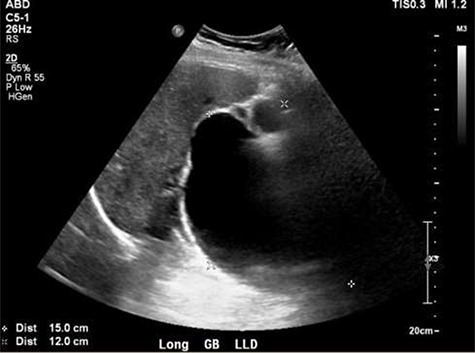
Ultrasound showing obscured CBD, 3 mm gallbladder wall, and a cystic lesion communicating with the CBD and a large gallbladder cyst.

CCs are classified based on the anatomical location, shape and extent of the cystic lesion in relationship to nearby structures [2, 3]. The size of choledochal cyst varies, but they rarely exceed 9 cm [4]. The classification is based on the Todani classification, which includes five subtypes of CCs [1, 2, 5] (Fig. 1). Type I CCs, which are the most common type are dilatation of the extrahepatic bile duct [6–8]. Type I CCs are further classified into cystic (IA), focal (IB) or fusiform (IC) [8]. Type II CCs are extrahepatic supra-duodenal diverticula of the common bile

**Figure 3:**
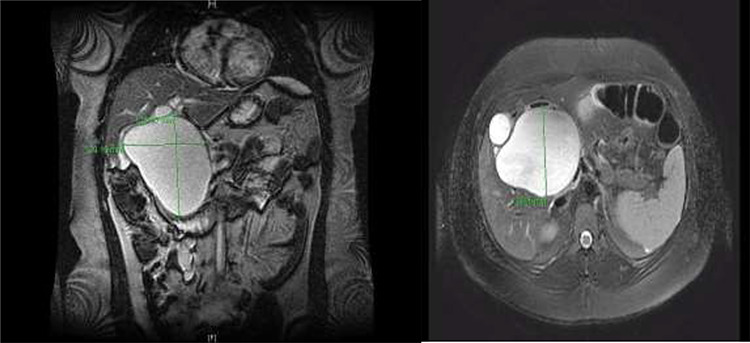
CC with mass effect on liver and duodenum in coronal (**A**) and axial (**B**) views.

duct [7]. Type III CCs are intra-duodenal diverticulum [3, 8]. Type IV CCs have intrahepatic and extrahepatic dilatation of the bile duct. Type IV is further divided into two subtypes: IVa which contains fusiform extrahepatic and intrahepatic cysts [3], and IVb which contains multiple extrahepatic cysts [3]. Type V CCs, also known as Caroli disease have multiple intrahepatic cysts [3, 5].

The exact etiology of CCs is unknown; however, delay in diagnosis and treatment CCs have been associated with a number of complications, including stone formation due to biliary stasis, inflammation, infections, pancreatitis, cholangitis and obstruction [3, 9] Additionally, malignancies of the bile duct and gall bladder are associated with CCs, with higher prevalence seen in types I and IV cysts [1]. One of the most critical steps in the management of a patient presenting with a CC is correct surgical planning based on an accurate classification of the CC [10]. Given the risks, delay in diagnosis can be detrimental. In this report, we discuss a case of a massive type I CC in an adult female to illustrate that although uncommon, these cysts can go untreated until they are very large, the importance of a through workup in diagnosing these entities, and success in treating them with proper surgical management.

## CASE PRESENTATION

An 18-year-old Caucasian female with BMI of 37 without past medical or surgical history was transferred to our hospital from for evaluation of right upper quadrant abdominal pain and jaundice. Laboratory values were notable for AST of 130 U/L (Ref. range: 10–36 U/L), ALT of 264 U/L (Ref. range: 7–35 U/L), total bilirubin of 2.6 mg/dL (Ref. range: 0–1.4 mg/dL), alkaline phosphatase of 235 IU/L (Ref. range: 44–147 IU/L), Glutamyl-Transferase (GGTP) level of 519 (Ref range: 9–64 U/L), which were concerning for a hepatobiliary etiology.

**Figure 4:**
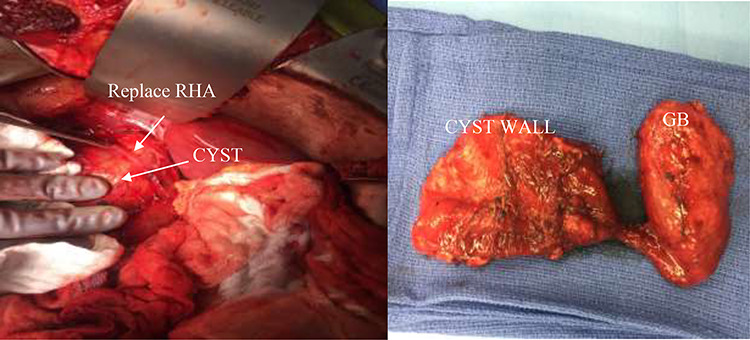
(Left) intra-operative CC and replace right hepatic artery. (Right) decompressed gallbladder (GB) and decompressed CC.

A right upper quadrant abdominal ultrasound was obtained which showed that the gallbladder wall was 3 mm, an obscured common bile duct (CBD), and a cystic lesion communicating with the CBD and a large gallbladder cyst (Fig. 2). A contrast MRI with magnetic resonance cholangiopancreatography (MRCP) of the abdomen revealed marked fusiform dilatation of the extrahepatic CBD measuring 8.8 cm anterior–posterior x 10.9 cm transverse x 12.7 cm craniocaudally, mild-to-moderate central intrahepatic bile duct dilatation and posterior displacement of the patent main portal vein due to associated mass effect (Fig. 3). Based on the image findings the diagnosis of a CC was made. The type, however, could not be definitively diagnosed as the cyst could be a large type I CC with resultant upstream intrahepatic ductal dilatation versus a type IVa CC with associated mild to moderate central intrahepatic biliary ductal dilatation. Upon review of the images, labs, assessment of the patient, she was scheduled for resection of the extra hepatic bile duct and choledochal cyst with planned Roux en y hepaticojejunostomy. Intraoperatively, she was found to have a massive 20 cm type I choledochal cyst (Fig. 4).

## DISCUSSION

CCs are congenital biliary duct anomalies. Types I and IV CCs are most prone to malignant transformation, but complete resection decreases this risk of transformation. Typically diagnosed and treated in childhood, they rarely present in adults. Symptomatology is vaguer in adults and this can often lead to delay the diagnosis and treatment. Although rare, CCs should be considered in the differential diagnosis in all patients with a history of biliary colic pain, and dilation or obstruction of the biliary tree. CCs are typically smaller than 9 cm, but in this case, we presented a massive 20 cm type I choledochal cyst in an adult. Timely diagnosis and definitive treatment of patients with CC yields a favorable prognosis and helps to prevent long-term complications associated with CC such as biliary cirrhosis, malignancy and portal hypertension. Therefore, clinicians should be familiar with proper diagnosis and treatment of these entities in both children and adults. Typically, ultrasound is the best initial method for evaluating the entire intrahepatic and extrahepatic biliary system and gallbladder. But, as shown in this case it does not always help in diagnosis especially with very large cysts. MRCP is the current gold standard in the imaging of CC and should be utilized accordingly.

Proper knowledge of these cysts, their varying presentations and their correct treatment is vital to surgeons. For our patient, as per the current surgical approach for Type I CC a complete cyst excision, cholecystectomy and bilioenteric reconstruction was performed utilizing the Roux-en-Y hepaticojejunostomy. The surgical treatment employed in this patient successfully resolved the biliary obstruction, restored normal biliary drainage and eliminated long-term risk for malignancy. This approach remains the gold standard. Due to their rare incidence, CCs are sometimes diagnosed intraoperative. It is important to be familiar with the proper diagnosis and management of these uncommon conditions to ensure optimal surgical treatment plane.

## CONFLICT OF INTEREST STATEMENT

None declared.

## FUNDING

None.
